# Stroke among Patients Admitted to a Tertiary Care Centre: A Descriptive Cross-sectional Study

**DOI:** 10.31729/jnma.8075

**Published:** 2023-04-30

**Authors:** Ram Narayan Kurmi, Laxman Mandal, Raj Singh, Prateek Kumar Chaudhary, Tirth Dhungana, Sandesh Lamichanne, Nimish Raj Bastola, Prajjwal Pokharel

**Affiliations:** 1 Maharajgunj Primary Health Centre, Maharajgunj, Kapilvastu, Nepal; 2 Department of Internal Medicine, Chitwan Medical College, Bharatpur, Chitwan, Nepal; 3 Kathmandu Model Hospital, Pradarshani Marg, Kathmandu, Nepal; 4 Gaushala Primary Health Centre, Gaushala, Mahottari, Nepal

**Keywords:** *hemorrhagic stroke*, *ischemic stroke*, *prevalence*

## Abstract

**Introduction:**

Stroke is the second most common cause of mortality after ischemic heart disease and the leading cause of morbidity around the world. This study aimed to find out the stroke among patients admitted to a tertiary care centre.

**Methods:**

A descriptive cross-sectional study was conducted in the Department of Internal Medicine and Neurosurgery from 15 July 2021 to 15 June 2022 after taking ethical approval from Institutional Review Committee (Reference number: 78/79-083). Convenience sampling method was used. Point estimate and 95% Confidence Interval were calculated.

**Results:**

Among 5034 patients, 149 (2.95%) (2.48-3.41, 95% Confidence Interval) patients had stroke. Out of 149 cases, male to female ratio was 1.06 with a mean age of 65.05±14.06 years. The most common presentation was hemiparesis 128 (85.90%). Hypertension 106 (71.14%) was the most common underlying condition. The frontal area 17 (32.02%) was the most common site of ischemic stroke. Putamen (55.26%) was most common site in hemorrhagic stroke. The mean hospital stays was 6.3±5.18 days. There were 5 (3.40%) cases of in-hospital mortality.

**Conclusions:**

The prevalence of stroke was similar to other studies done in similar settings.

## INTRODUCTION

According to WHO, stroke is defined as "The rapidly developing clinical symptoms and signs of focal (at times global) disturbance of cerebral function with symptoms lasting more than 24 hours or leading to death with no apparent cause other than that of vascular origin". Stroke leads to long-term disability and a high economic burden to the family and society.^1^

In Nepal, among the total patients admitted to hospitals, 2.25% are stroke patients.^2^ Stroke is the 2nd most common cause of mortality and the leading cause of morbidity in the world.^3^ Hemiplegia is the most common presentation of stroke. Ischemic stroke accounts for 50-85% of total strokes in the world. Hypertension, diabetes mellitus, dyslipidemia, and smoking were the most common modifiable risk factor for stroke.^4^

The aims of this study was to find out the stroke among patients admitted to a tertiary care centre.

## METHODS

This was the descriptive cross-sectional study conducted in the Department of Internal Medicine and Neurosurgery in Chitwan Medical College Teaching Hospital (CMCTH), from 15 July 2021 to 15 June 2022. The study was done after ethical approval from the Institutional Review Committee (IRC) of CMCTH (Reference number: 78/79-083). All the patients above age 18 yrs and having clinical and computed tomography (CT) confirmed diagnosis of stoke was included in the study. Patients with incomplete medical record, patients with transient ischemic attack (TIA), and patients who leave the hospital against medical advice were excluded from the study. A convenience sampling was used. Sample size was calculated by using the following formula:


$$\begin{array}{ll} \text{n} & ={\text{Z}}^{2}\times \frac{\text{p}\times \text{q}}{{\text{e}}^{\text{2}}}\\ & =1.96^{2}\times \frac{0.50\times 0.50}{0.02^{2}}\\ & =2401 \end{array}$$

Where,

n = minimum required sample size
Z = 1.96 at 95% Confidence Interval (CI)
p = prevalence taken as 50% for maximum sample size
q = 1-p
e = margin of error, 2%

The calculated sample size was 2401. After doubling sample size, the total sample size was 4802. However, total 5034 sample size was taken.

Dyslipidemia was defined as the presence of any of the following: patients on lipid-lowering drugs or total cholesterol >240 mg/dl, triglycerides (TG) >150 mg/dl, low-density lipoprotein >130 mg/dl, and high-density lipoproteins (HDL) <50 mg/dl for female and <40 mg/dl for male. Diabetes Mellitus was defined as symptoms of diabetes, random blood sugar >200 mg/dl or HbA1C level >6.5 or if a patient was on oral hypoglycemic agents. Hypertension was defined as systolic blood pressure >140 and/or diastolic >90 mmHg and/or on anti-hypertensive treatment.^5,6^

Data were collected retrospectively from medical records 15 July 2021 to 15 June 2022. The age, sex, Glassgow Coma (GCS) at admission, blood pressure, clinical presentation, comorbidities, risk factors, family history, past history of stroke, anticoagulant drugs, lipid profiles, random blood sugar, and radiological findings were collected from patients' case note from the medical department of the hospital and electronic data registry system (Midas) in the proforma.

Data were entered in Microsoft Excel Version 2010 and analyzed using IBM SPSS Statistics version 21.0. Point estimate and 95% CI were calculated.

## RESULTS

Among 5034 patients, 149 (2.95%) (2.48-3.42, 95% CI) had a stroke. Among these, 46 (30.87%) cases were from Neurosurgery Department and 103 (69.12%) cases were from Internal Medicine Department. A total of 77 (51.67%) patients were males and 72 (48.32%) were females with a ratio of 1.069. Patients' ages range from 32 to 96 years with a mean age of 65.05±14.06 years. CT findings showed 103 (69.12%) had ischemic stroke whereas 46 (30.87%) had a hemorrhagic stroke. A total of 89 (59.73%) patients had left-sided strokes. Out of which 60 (67.41%) had ischemic stroke and 29 (32.58%) had a hemorrhagic stroke. The mean age of patients with hemorrhagic stroke was 61.20±14.29 years and those with ischemic stroke were 66.78±13.67 years (Table 1).

**Table 1:** Age-wise distribution of stroke (n= 149).

**Type of Stroke**		
Age (years)	Ischemic n (%)	Hemorrhagic n (%)
31-40	4 (2.68)	2 (1.34)
41-50	13 (8.72)	11 (7.38)
51-60	11 (7.38)	10 (6.71)
61-70	33 (22.14)	8 (5.36)
71-80	27 (10.06)	12 (8.05)
>80	15 (10.06)	17 (34)

GCS below 8 was found in 10 (6.71%) patient with stroke (Table 2).

**Table 2:** AGCS at the time of presentation to the hospital (n= 149).

GCS	Ischemic n (%)	Hemorrhagic n (%)
13-15	85 (57.04)	26 (17.45)
9-12	14 (9.39)	14 (9.39)
3-8	4 (2.68)	6 (4.02)

The most common presentation was hemiparesis which was 128 (85.90%) followed by slurred speech 73 (49%) and facial deviation 59 (39.59%) (Table 3).

**Table 3:** Clinical presentation of stroke patients (n= 149).

Clinical Presentation	Ischemic n (%)	Hemorrhagic n (%)
Hemiparesis	96 (64.42)	32 (21.47)
Slurred Speech	52 (34.89)	21 (14.09)
Facial Deviation	49 (32.88)	10 (6.71)
Decrease consciousness	16 (10.73)	11 (7.38)
Vomiting	11 (7.38)	16 (10.73)
Aphasia	20 (13.42)	17 (34)
Headache	12 (8.05)	7 (7.69)
Loss of consciousness	8 (5.36)	7 (7.69)
Seizure	2 (1.34)	6 (4.02)

Of the total 149 patients, the most common underlying condition was hypertension 106 (71.14%) followed by dyslipidemia 47 (31.54%) and diabetes mellitus 31 (20.80%). The mean systolic and diastolic blood pressure for hemorrhagic stroke was 168.04±28.64 mmHg and 99.35±12.89 mmHg. The mean systolic and diastolic blood pressure for ischemic stroke was 147.67±31.629 mmHg and 87.77±15.01 mmHg respectively (Table 4).

**Table 4:** Underlying condition (n= 149).

Underlying condition	n (%)
Hypertension	106 (71.14)
Dyslipidemia	47 (31.54)
Diabetes mellitus	31 (20.80)
Alcohol	28 (18.79)
Smoking	24 (16.10)
Anticoagulant drug	17 (11.40)
Atrial fibrillation	15 (10.06)
Past history of stroke	15 (10.06)

In our study, the most common site of hemorrhagic stroke was the putamen 21 (55.26%) followed by the thalamus 7 (18.42%). Similarly, the most common site of ischemic stroke was the frontal region 17 (32.05%) followed by basal ganglia 10 (18.86%). The mean hospital stays was 6.3±5.18 days. There were 5 (3.4%) cases of in-hospital mortality. Of which, 3 (2.01%) died due to ischemic stroke and 2 (1.34%) with hemorrhagic stroke (Table 5).

**Table 5:** CT findings.

	Ischemic stroke (n= 53).	Hemorrhagic stroke (n= 38).
Site of lesion	n (%)	n (%)
Putamen	2 (3.70)	21 (55.26)
Thalamus	-	7 (18.42)
Basal ganglia	10 (18.86)	1 (2.6)
Frontal	17 (32.02)	-
Parietal	8 (15.09)	4 (10.5)
Temporal	5 (9.43)	-
Occipital	2 (3.77)	1 (2.6)
Cerebellar	2 (3.77)	2 (5.2)
Brainstem	2 (3.77)	1 (2.6)
Ventricle	-	1 (2.6)
Internal capsule	4 (7.54)	-
Medulla	1 (1.88)	-

## DISCUSSION

In our study, the prevalence of stroke was 2.95% which is similar to the other studies.^2^ The incidence of stroke is increasing.^4^ The mean age of stroke was 65.05 with most patients lying within the range of 61-70 years. A comparable age was also present in studies done in Nepal and India.^7^'^13^

In our study, patients aged less than 40 years comprised 10.7% of the total patients which was comparable to the study done in Yemen (13.6%), and Nepal (11.74%).^14,15^

Ischemic strokes account for 50-85% of all strokes worldwide.^4^ The most common type of stroke in our study was ischemic (61%) which was similar to the other study done in Nepal.^16,17^ However, a population-based study done in neighbouring country China had an ischemic stroke of 91.7% which was attributed due to well-controlled hypertension among the population.^18^

Clinical presentation of stroke depends on the type of stroke and artery involvement which supplies specific areas of the brain. In our study, the most common presentation was hemiparesis (85.9%) followed by slurred speech (49%). Similarly, a study done in Kathmandu had hemiparesis (90.2%) followed by slurring of speech (33.33%) as the most common presentation.^9^

As per the study, ten risk factor was associated with a 90% risk of stroke. In our study, hypertension (71.14%) was the most common modifiable risk factor followed by dyslipidemia (31.54%). Some studies done in Nepal had hypertension (61.2%, 50%, 42%, and 48.7% respectively) as the most common modifiable risk factor which was followed by smoking (59.4%, 35.1%, 28.5%, and 41.5% respectively).^7,11,17,19^ However, another study done in Nepal revealed smoking (60%, 58%, 61%, and 48%, respectively) as the most common risk factor.^2,9,10,15^ The relatively low prevalence of smoking 24 (16.10%) in our study might be due to the failure to record the smoking history of the patients. However, dyslipidemia accounts second most common risk factor in our study which is probably due to changes in the dietary habit of Nepalese. A study done in China had dyslipidemia as a major risk factor with a prevalence of 57.1%.^18^ This revealed that smoking and dyslipidemia are the most important modifiable risk factor of stroke in the context of Nepal after hypertension. The INTERSTROKE study showed atrial fibrillation leads to 9% of the ischemic stroke with variation among different regions; high-income countries (23%), South America (13%), Africa (7%), India (6%), and Southeast Asia (5%).^3^ Atrial fibrillation was present in 10.06% which was comparable to another study done in Nepal with a prevalence of 12.5%, 8%, and 9%, respectively.^9,10,15^ In this study,10% of total patients had a history of stroke which was comparable to another study done in Nepal (8.3%)^11^ and Yemen (12.2%).^14^

In our study, the most common site of ischemic stroke was the parietal (32.05%) followed by basal ganglia (18.86%) which was consistent with the study done in India with parietal (41%) most followed by basal ganglia (16%).^13^ Similarly, the most common site of hemorrhagic stroke in our case was the putamen (55.26%) followed by the thalamus (18.42%) however, basal ganglia (43.9%) was followed by the thalamus (14.61%) in the same study done in India as the most common site of bleeding.^13^

The hospital stays range from 1 to 36 days with a mean stay of 6.3 days. Similarly, a study done in Nepal also stated the range of 1 to 33 days with a mean of 6.11 days.^11^ Another study was done in a similar setting also had a higher hospital stay for hemorrhagic stroke(10±9.4 days) than ischemic stroke (7.1 ±8.1 days).^19^ In our study, the in-hospital mortality rate was 3.4% which was similar to another study done in Nepal (5.7%).^2^

Our study could not find a significant association between the majority of risk factors, clinical presentation and laboratory values. This was a singlecentred based descriptive cross-sectional study so a more robust, extensive and multicentered prospective study should be done to address the magnitude of disease and its association with different variables in the context of the Nepalese population.

## CONCLUSIONS

The prevalence of stroke was similar to other studies done in similar settings. Stroke is a public health problem with male predominance. Though stroke was regarded as a disease of old age, its prevalence in young patients is on an increasing trend. Health policymakers must emphasize effective preventive measures for stroke and its modifiable risk factors (hypertension, diabetes mellitus, dyslipidemia, smoking) to decrease mortality, morbidity and economic burden among the patients as well as in the nation.
